# A rare case of trigeminal trophic syndrome with periorbital cellulitis and full-thickness upper eyelid defect in an undiagnosed patient with human immunodeficiency virus: a case report

**DOI:** 10.1186/s13256-024-04621-0

**Published:** 2024-07-22

**Authors:** Stephen Apanga, Mohammed Alhassan, Bawa Abdulai

**Affiliations:** 1 Department of Community Health and Preventive Medicine, School of Medicine, University for Development Studies, Tamale, Ghana; 2 Yizura Hospital Limited, Kintampo, Ghana

**Keywords:** Trigeminal trophic syndrome, HIV, Periorbital cellulitis, Full-thickness upper eyelid defect

## Abstract

**Background:**

Trigeminal trophic syndrome is a rare cranial and facial condition caused by damage to the central or peripheral branches of the trigeminal nerve. This syndrome consists of a triad of anesthesia, paresthesia, and crescent-shaped facial ulcer involving the ala nasi and sometimes extending to the upper lip. Although previous screening for human immunodeficiency virus in some patients with trigeminal trophic syndrome was negative, we present a unique case of trigeminal trophic syndrome who tested positive for human immunodeficiency virus with eye complications.

**Case presentation:**

We present a rare case of trigeminal trophic syndrome in a 44-year-old Black African woman who tested positive for human immunodeficiency virus. She presented with a 6-week history of progressive, persistent, and painless left sided facial and scalp ulcerations that started as small skin erosion. Diagnosis of trigeminal trophic syndrome was made on clinical grounds based on the triad of anesthesia, paresthesia, and unilateral crescent-shaped ulcer in the trigeminal dermatome and her past medical history. The ulcer healed completely after counseling and pharmacological therapy, but she later developed left periorbital cellulitis and left upper eyelid full-thickness defect.

**Conclusion:**

This is by far the first documented case of trigeminal trophic syndrome with a positive human immunodeficiency virus test. Testing for human immunodeficiency virus in patients with trigeminal trophic syndrome is necessary as this can help improve clinical management and treatment outcomes. Seeking the services of specialists remotely in resource constraint settings is beneficial for managing complications associated with trigeminal trophic syndrome.

## Background

Trigeminal trophic syndrome (TTS) is a rare facial and cranial condition resulting from injury or damage to the central or peripheral branches of the trigeminal nerve [1–4]. The syndrome consists of a triad of anesthesia or hypoesthesia, paresthesia, and frequently crescent-shaped neurotrophic facial ulceration in the trigeminal dermatome that is persistent or recurrent in nature [1, 4–7], mostly involving the ala nasi and sometimes extending to the upper lip [1, 2, 5, 7–9]. The paresthesia in TTS often presents as a burning, itching, crawling, or tingling sensation with picking or rubbing of the affected area sometimes [1, 6, 9–11]. The most common causes of TTS are iatrogenic: mainly from therapeutic procedures such as trigeminal rhizotomy and trigeminal nerve ablation through alcohol injection of the Gasserian ganglion and coagulation [1, 2, 5, 9, 11]. Other associated causes include stroke, brain tumors (astrocytoma and meningioma), infections (herpes, syphilis, and leprosy), trauma, craniotomy, and other unknown causes [1, 5, 9–12].

There is currently no specific algorithm for diagnosing TTS, and therefore, its diagnosis is based mainly on clinical grounds and past neurological history. Laboratory and biopsy findings are often either normal or inconclusive [1, 9, 11]. Although previous screening for human immunodeficiency virus (HIV) in some patients with TTS was negative, here we report a rare case of a 44-year-old woman with TTS who tested positive for HIV complicated by periorbital cellulitis and left upper eyelid full-thickness defect.

## Case presentation

A 44-year-old Black African woman came to the outpatient clinic with a 6-week history of progressive, persistent, painless left-sided facial, and scalp ulcerations that started as small skin erosion. She had previously visited a health facility 2 weeks after the ulcer started and was diagnosed with septic and allergic dermatitis as a result of persistent itching of the affected area. The patient admitted to frequent manipulation of the area due to tingling and crawling sensation and later loss of sensation as well. Her past medical history indicated the absence of brain surgery, hypertension, stroke, diabetes, herpes and syphilis infections, and head trauma. She also indicated that, because of the fear of stigma due to the ulcer, she had to stay away from a number of social activities, which got her depressed.

**Fig. 1:**
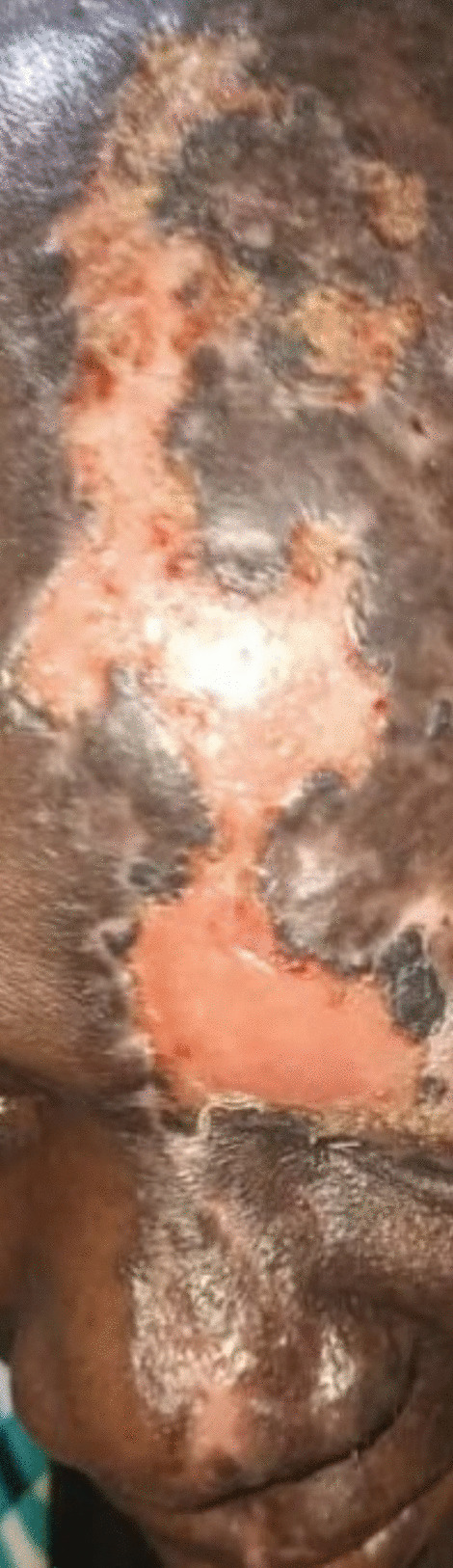
Left-sided deep ulceration of the forehead and scalp involving the upper eyebrow and extending to the bridge of the nose, ala nasi, and upper lip

On physical examination, she appeared healthy with a temperature of 36.0 °C, weight of 75 kg, and blood pressure of 110/80 mmHg. Dermatological examination revealed a left-sided deep ulcer involving the upper eyebrow and extending to the bridge of the nose, ala nasi, upper lip, forehead, and scalp along the distribution of the ophthalmic branch of the trigeminal nerve (Fig. 1). Neurological examination revealed decreased pain and loss of sensation over the ulcerated areas. Examination findings from other systems were normal.

Laboratory investigations that included full blood count, blood glucose test, and syphilis test were all normal. However, serological HIV testing was reactive. Chest X-ray findings were normal. She declined to have a computed tomography (CT) scan done because this required her to travel out of the community to a higher center in a different town to have it done.

**Fig. 2:**
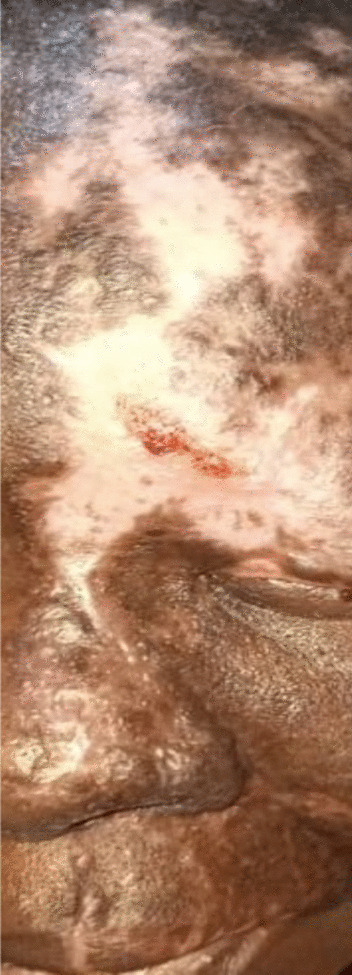
Healing of ulceration 2 weeks after commencement of treatment

A diagnosis of TTS was made on clinical grounds based on the triad of anesthesia, paresthesia, and the unilateral crescent-shaped frontal ulceration in the trigeminal dermatome and her past medical history. She was started on amitriptyline 25 mg at night for 4 weeks and oral antibiotic flucloxacillin 500 mg four times daily for 1 week and was counseled on the need to stop picking and rubbing the affected area. She was then referred to the district antiretroviral clinic where she was put on only a daily dose combination of dolutegravir (as sodium) 50 mg/lamivudine 300 mg/tenofovir disoproxil fumarate 300 mg after 1 week of referral. During a clinic follow-up 2 weeks after commencing treatment, the ulcer was healing (Fig. 2) and she was glad with the healing process especially after starting the antiretroviral therapy.

**Fig. 3:**
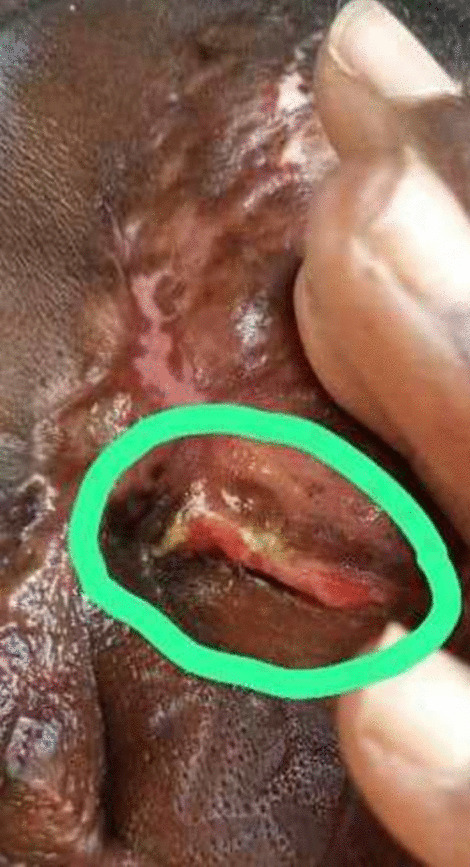
Periorbital edema, erythema, and purulent discharge (green circle) of the left upper eye lid

On 2-month clinic follow-up, the ulcer was healing well with hair growth. When a follow-up visit was made a month later, the ulcer had completely healed with formation of keloids and hair growth. However, there was left eye periorbital edema and erythema associated with purulent discharge (Fig. 3) with no visual impairment. She declined a referral to see an ophthalmologist for further evaluation and treatment. A diagnosis of periorbital cellulitis was reached after consultation with an ophthalmologist through pictures. Oral antibiotics of amoxicillin + clavulanic acid 625 mg twice daily for 1 week and azithromycin 500 mg daily for 6 days were prescribed. In a 9-month follow-up after initial presentation, there was left upper eyelid full thickening requiring the use of her hand to part the eyelid anytime she wanted to open her left eye. The ulcer had completely healed with no recurrence, and she was very appreciative of the efforts of the clinical team especially by involving her family. However, all efforts are being put in place by the clinical team to arrange for reconstructive surgery for her upper eyelid defect.

## Discussion

TTS is a rare facial and cranial condition that results from injury or damage to the central or peripheral branches of the trigeminal nerve [1–4]. This rare condition has been found to be most common among women than men [4, 5]. The syndrome presents with a classical clinical triad of anesthesia or hypoesthesia, paresthesia, and frequently crescent-shaped facial ulcer mostly involving the ala nasi and sometimes extending to the upper lip [1, 2, 4–9]. Although the mechanism of skin ulceration is unclear, it is believed to be due to altered skin sensations such as numbness, tingling and pricking from the affected injured nerves leading to self-induced mutilation or trauma of the skin [1, 3, 10, 13].

Therapeutic procedures such as trigeminal rhizotomy and trigeminal nerve ablation through alcohol injection of the Gasserian ganglion and coagulation [1, 2, 5, 9, 11] have been implicated to be the main causes of TTS. However, Wallenberg syndrome (stroke/vascular insufficiency), brain tumors (astrocytoma and meningioma), infections (herpes, syphilis, and leprosy), trauma, craniotomy, and other unknown causes [1, 5, 9–12] have also been found to be commonly associated with TTS. The diagnosis of TTS is often based on clinical grounds through the presence of the clinical triad of anesthesia, paresthesia, and crescent-shaped facial ulcer and sometimes past neurological history [1, 4–7]. Laboratory and biopsy findings have not been beneficial in diagnosing TTS as they are either often normal or inconclusive [1, 9, 11] but are nonetheless important in excluding its differential diagnoses. Differential diagnoses of TTS include other causes of ulcerations such as skin neoplasms (basal cell carcinoma, squamous cell carcinoma, malignant lymphoma, sarcoma), systemic vasculitis (Wegener’s granulomatosis), infection (herpes, syphilis, mycobacteria, dimorphic fungi, varicella, leishmaniasis), granulomatous disease, pyoderma gangrenosum, midline granuloma of the face, and facial dermatitis [2–4, 9, 11]. In our case, the diagnosis of TTS was arrived at solely on clinical grounds after ruling out some differential diagnoses from her previous history and carrying out some laboratory investigations. Our inability to investigate neoplasms as a possible cause of the facial ulcer by carrying out a CT scan was not only complicated by the patient’s refusal to have a scan done but was also due to limited diagnostic equipment and capabilities in a resource constraint setting such as ours. Unlike in previous few cases where HIV testing was done and found to be negative [4, 7], our patient tested positive for HIV, thereby making our case the first to be reported, to the best of our knowledge, and a unique one as well. Though one of the main causes of TTS is trigeminal nerve ablation with rhizotomy and/or nerve block methods for treatment of trigeminal neuralgia, no such neuralgic case was found to be a complication of HIV infection [1, 2, 5, 9, 11].

The management of TTS can be challenging as there is no established treatment protocol. However, managing this syndrome requires a multidisciplinary approach involving behavioral modification, wound care, pharmacological treatment, and surgical intervention when necessary. Behavioral modification requires that patients be educated and counseled on the need to avoid the self-induced mutilation of the skin resulting from repetitive picking or rubbing of the affected area [1, 3, 6, 9, 11, 12]. Wound care is essential in the healing process of ulcers associated with TTS and sometimes in reducing further trauma. These include procedures such as application of occlusive dressings [1, 2, 12]; hydrocolloid dressings [1, 2, 4, 9]; application of thermoplastic dressings [1, 7, 14]; negative pressure wound therapy and application of vacuum dressings [4, 6, 14, 15]; and the use of antibiotics [1, 9, 11, 12]. Pharmacological or medical management, though with varying therapeutic outcomes, often involves the use of medications such as carbamazepine, pregabalin, gabapentin, amitriptyline, pimozide, chlorpromazine, benzodiazepines, topical tacrolimus, vitamin B supplementation, and acyclovir, among others [1, 3, 4, 6, 7, 9–11]. Similarly, there have been reports of the use of surgical methods that include procedures including surgical reconstruction with local and regional flaps [1, 3, 9, 10]; construction of prosthesis [11]; and cervical sympathectomy and transcutaneous electrical nerve stimulation to improve blood supply resulting in wound healing [1, 9, 10]. Surgical interventions have, however, often resulted in varied degrees of success, with most cases having recurrence of ulcers due to continuous self-manipulation of the skin. For our patient, a combination of behavioral modification, wound care, and pharmacological treatment resulted in complete healing of the ulcer within 3 months.

Other comorbidities have been found to complicate TTS. People with TTS often have psychiatric comorbidities such as anxiety, obsessive–compulsive disorder, mood dysfunction [12], and Alzheimer’s disease [1, 3, 7], thereby requiring psychiatric or psychological evaluation as a management modality. This patient appeared depressed and withdrew from most social events because of the fear of stigma emanating from her facial ulcer. Due to the absence of psychiatric or psychological services in this setting, coupled with her refusal for referral, the clinical team had to counsel her and her family. Counseling was also supported by regular home visits as a means of providing her with the necessary psychological support. Ophthalmic conditions such as orbital cellulitis [12], corneal lesions [1, 6, 7], eyelid or canthal lesions, and eyelid defects [1, 6] have also been observed to complicate TTS, hence the need for ophthalmological review in some cases. In our case, ophthalmological review was achieved by engaging the services of an ophthalmologist through the use of photos at each stage of eye care.

## Conclusion

We presented a rare case of TTS resulting in a complication of periorbital cellulitis and full-thickness upper eyelid defect in an HIV-positive woman who was previously undiagnosed. Although the outcome of HIV testing was negative in previous cases of TTS, it is important to test for HIV to ensure that HIV-positive patients are put on antiretroviral therapy early as this can help improve treatment outcomes. It is necessary for clinicians in resource-constrained settings to explore the option of engaging specialists through the use of phones or pictures to assist them in managing the complications of TTS when patients refuse referrals for whatever reason.

## Data Availability

The authors confirm that the data supporting the findings of this study are available within the article.

## Abbreviations

CT: Computer tomography
HIV: Human immunodeficiency virus
TTS: Trigeminal trophic syndrome
